# Dyslipidemia and its associated factors in southern Iranian women, Bandare-Kong Cohort study, a cross-sectional survey

**DOI:** 10.1038/s41598-021-88680-z

**Published:** 2021-04-28

**Authors:** Marzieh Nikparvar, Mohadeseh Khaladeh, Hadi Yousefi, Mohammadsadegh Vahidi Farashah, Behzad Moayedi, Masoumeh Kheirandish

**Affiliations:** 1 Cardiovascular Research Center, Hormozgan University of Medical Sciences, Bandar Abbas, Iran; 2 Student Research Committee, Faculty of Medicine, Hormozgan University of Medical Sciences, Bandar Abbas, Iran; 3 Department of Epidemiology and Biostatistics, Faculty of Medicine, Hormozgan University of Medical Sciences, Bandar Abbas, Iran; 4 Endocrinology and Metabolism Research Center, Hormozgan University of Medical Sciences, Shahid Mohammadi Hospital, Jomhuri Eslami Blvd, Bandar Abbas, 7919915519 Iran; 5 Soft Computing Lab, Department of Computer Engineering, Amirkabir University of Technology, Tehran, Iran

**Keywords:** Diseases, Health care

## Abstract

Dyslipidemia, a major risk factor for cardiovascular diseases, has become a global issue. Due to the variations in the prevalence of dyslipidemia, this study aimed to evaluate dyslipidemia and its associated factors in women of the Bandare-Kong Cohort Study (BKNCD). This study was conducted on women from the population-based BKNCD, as part of the Prospective Epidemiological Research Studies in IrAN (PERSIAN). Sociodemographic data, medical history, and anthropometric indices were collected. Dyslipidemia was defined as any lipid abnormality including low-density lipoprotein (LDL) ≥ 160, total cholesterol (TC) ≥ 240, high-density lipoprotein (HDL) < 40, or triglyceride > 200 mg/dl. From the 2223 women in this study (mean age: 48.28 ± 9.26 years), dyslipidemia was observed in 851 (38.3%). High TC was the most common lipid abnormality (18.5%) followed by high LDL (17.7%). Dyslipidemia was most prevalent among women aged 55–70 years, the married, those with < 6 years of education, the unemployed, the overweight or obese, with low socioeconomic status, diabetes, hypertension, and high waist circumference, those using the hookah and living in urban areas. Logistic regression revealed that women with high waist-to-hip ratio (WHR) (OR = 2.22, 95% CI 1.60–3.08), those aged 45–54 years (OR = 1.34, 95% CI 1.07–1.68) and 55–70 years (OR = 1.33, 95% CI 1.03–1.72), and those living in urban areas (OR = 1.35, 95% CI 1.05–1.73) were at significantly increased risk of dyslipidemia. In addition, the results were confirmed using deep neural network models. Dyslipidemia was highly prevalent in Iranian women in the southern coastal region. Central obesity, age over 45 years, and living in urban areas appear to be relatively significant risk factors for dyslipidemia among women.

## Introduction

Dyslipidemia, a disorder of lipid metabolism, is clinically defined as the presence of one of the following abnormalities: elevated plasma triglycerides (TG), elevated total cholesterol (TC), high levels of low-density lipoprotein (LDL), and decreased high-density lipoprotein (HDL)^1^. With the increasing prevalence of dyslipidemia, mostly due to adverse changes in lifestyle including dietary changes, the more sedentary lifestyle, and reduced physical activity, it has become a global public health issue^2^. There is substantial evidence that dyslipidemia is associated with an increased risk of cardiovascular disease (CVD)^3^. According to the World Health Organization (WHO) estimates, dyslipidemia, especially high TC, is responsible for 2.6 million deaths annually and 29.7 million disability-adjusted life years (DALYS) worldwide^4^.

The prevalence of dyslipidemia varies in different regions, with hypercholesterolemia ranging from 22.6% to 54% across Africa, South East Asia, Europe, and America^4^. Studies in Iran have also reported the prevalence of dyslipidemia: the prevalence of hypertriglyceridemia, hypercholesterolemia, high LDL, and low HDL ranged 14–40.6%, 14–61%, 13.4–45.5%, and 5–73%, respectively^5–8^. In addition, it has been demonstrated in many parts of the world including Iran, that dyslipidemia can be influenced by numerous factors including socio-economic status, level of fat intake, obesity, and gender^9–11^. Dyslipidemia is a modifiable risk factor for the development of type 2 diabetes, atherosclerosis, CVD, and stroke; early effective management of patients with dyslipidemia can decrease the incidence and the burden of the above-mentioned conditions^12–14^.

Metabolism in women can be affected by alterations in hormonal levels throughout their lives, in either the premenopausal or postmenopausal period^15^. It has been reported that serum TC levels in women increases with age; however, this occurs more gradually compared to men^10^. Yet, it increases at a higher rate after the age of 44 years, probably as a result of the loss of estrogen in the postmenopausal period and decreased activity of LDL receptors^16^.

With regard to regional differences in the prevalence of dyslipidemia and gender variations in this respect, and taken into consideration the modifiable nature of dyslipidemia for prevention and control of the disease burden, as well as specific hormonal effects in females, it would be extremely important to be aware of the prevalence and potential influencing factors of this condition in women. Indeed, there is no information about the women who live in the southern coastal of Iran, thus we aimed to evaluate dyslipidemia and its associated factors in women of the PERSIAN Bandare-Kong Cohort Study.

## Methods

### Participants

We evaluated the women of the PERSIAN Bandare Kong Cohort Study, a prospective, population-based cohort study in Bandare-Kong, Iran, which has been previously described in detail^17^. This cohort study includes 2334 women aged 35–70 years, recruited between November 17, 2016, and November 22, 2018, from Hormozgan province, southern Iran, as part of the Prospective Epidemiological Research Studies in IrAN (PERSIAN). Written informed consent was obtained from all the participants. After the exclusion of pregnant women, those taking lipid-lowering medications, and incomplete records, 2223 women were included in the final analysis. All methods were carried out in accordance with relevant guidelines and regulations.

### Study design

The BKNCD cohort study is part of the PERSIAN (Prospective Epidemiological Research Studies in IrAN) Cohort. Sociodemographic data were collected using a face-to-face interview by trained interviewers. Age, education, marital status, place of residence, and hookah use were recorded. Data regarding occupation, type of residence ownership, home size/area, trips, and other possessions including cars, computers, dishwashers, etc. were used to determine the socioeconomic status (SES) by means of principal component analysis. Daily calorie intake was calculated using daily ingested foods reported by the participants and their calorie content. Daily and weekly energy expenditure were determined using the metabolic equivalent of tasks (METs).

Weight was measured with a digital scale (measurement accuracy of 0.5 kg), with subjects in minimum clothing and without shoes. Height was measured with subjects standing shoeless and with their shoulders set normally. Waist circumference (WC) was measured twice for each participant and the average was recorded. WC was measured at the end of several consecutive natural breaths, at a level parallel to the floor, the midpoint between the top of the iliac crest and the inferior margin of the last palpable rib in the midaxillary line. Hip circumference (HC) was measured at the largest circumference of the buttocks, at a parallel level to the floor. All measurements were done with the same stretch-resistant tape to the nearest 0.5 cm. Subjects were standing upright during the measurements, with arms relaxed at the side, feet evenly spread apart and body weight evenly distributed. Waist-to-hip ratio (WHR) was calculated as WC divided by HC to the nearest 0.01. WHO cut-off for substantially increased risk of metabolic complications in women are: WC > 88 cm and WHR ≥ 0.85. According to the study by Azizi et al. the cut-off value of WC for the Iranian population is WC ≥ 95 cm for both men and women^18^. No WHR cut-off has been established for the Iranian population; therefore, the WHO cut-off for WHR was used.

BMI was calculated as weight in kilograms divided by the square of the person’s height in meters to the nearest 0.01. Participants were categorized into two groups: BMI < 25 kg/m^2^ and BMI ≥ 25 kg/m^2^.

Blood pressure (BP) was measured using a standard mercury sphygmomanometer after 5 min of rest with an appropriate cuff size for the upper-arm circumference, in the seated position, with feet on the floor, and arm supported at heart level. The average of two measurements made at least 5 min apart was used for analysis. Hypertension was defined as sustained blood pressure ≥ 140/90 mmHg or treatment with anti-hypertensive medications. Elevated values (≥ 140/90 mmHg) were confirmed on a separate day.

Venous blood samples were collected following overnight 8-h fasting and fasting plasma glucose (FPG) was measured. Plasma glucose measurements were done using the glucose oxidase method. According to the American Diabetes Association (ADA) criteria, diabetes was defined as an FPG ≥ 126 mg/dl, confirmed in a repeat test, or treatment with glucose-lowering agents. Venous blood samples were collected on another day following overnight 12-h fasting and TC, TG, LDL, and HDL were measured for each participant using the enzymatic method. LDL < 100 mg/dl was considered optimal, 100–129 mg/dl near or above optimal, 130–159 borderline high, 160–189 high, and ≥ 190 very high. TC < 200 mg/dl was considered desirable, 200–239 borderline high, and ≥ 240 high. Low HDL was defined as HDL < 50 mg/dl in women and HDL ≥ 60 mg/dl was considered high. TG < 150 mg/dl was considered normal, 150–199 borderline high, 200–499 high, and ≥ 500 very high. Based on the following criteria, dyslipidemia was defined as the presence of one or more of the following disorders^19^:

1. TC ≥ 240 mg/dl
2. TG > 200 mg/dl
3. LDL ≥ 160 mg/dl
4. HDL < 40 mg/dl (in women)

### Data analysis

The Statistical Package for the Social Sciences (SPSS) software (version 25.0, Armonk, NY: IBM Corp.) was used for data analysis. Mean, standard deviation, frequency, and percentages were used to describe the results. The binary logistic regression model was used to examine the correlation of dyslipidemia and its components with the associated factors. Qualitative variables with P-values ≤ 0.2 in single correlations by the logistic regression were included in the general model. Area under the receiver operating characteristic (AUROC) curve was calculated to determine the prediction performance of the logistic regression model^20^. Linear regression was used to determine the predictive power of associated factors on lipid profile components. All potential predictive variables of quantitative nature were included in the linear regression model. P-values of equal to or less than 0.05 were regarded as statistically significant.

The deep neural network model (deep learning) was used for complementary analysis. Currently, different types of deep learning analysis are widely used whether in image analysis or discrete value analysis such as patients’ information. The analysis of tumor detection and classification in Alzheimer’s disease are two example of MRI image analysis that use deep convolutional neural networks (CNN)^21^. Based on the information and desired analysis, different deep learning methods can be used in a model and deep feed forward neural network is used for our approach. The model consists of a deep feed forward neural network with 9 layers using Python programming. Based on common practice, we split the data into 70% as a training and 30% as a testing set. The methods of calculating the accuracy, precision, recall, and classification error are shown in equations. Precision = (TP)/(TP + FP). In this equation, true positive (TP) represents transactions that were positive and classified as positive. True negative (TN) represents the number of transactions that were negative and classified as positive. False positive (FP) also indicates the number of transactions that were positive and classified as negative. Finally, FN (False Negative) shows transactions that were negative and classified as negative. The equation to the validity and recall assessment is as follows: Recall = (TP)/(TP + FN)^22^. The F1 score is the harmonic mean of the precision and recall. The highest possible value of an F-score is 1.0, indicating perfect precision and recall, and the lowest possible value is zero, when either the precision or the recall is zero.

### Ethics approval and consent to participate

The cohort study was given ethical approval by the Ethics Committee of Hormozgan University of Medical Sciences.

## Results

From the 2223 women evaluated in this study (with the mean age of 48.28 ± 9.26), dyslipidemia was observed in 851 (38.3%). The mean values of TC, TG, HDL, and LDL were 204.72 ± 42.39 mg/dl, 127.19 ± 68.73 mg/dl, 50.37 ± 10.83 mg/dl, and 129.16 ± 35.52 mg/dl, respectively. High TC was the most common lipid abnormality (18.5%) followed by high LDL (17.7%), low HDL (14.8%), and high TG (10.8%). One, two, three, and four abnormal lipid components were observed in 18.4%, 16.5%, 3.1%, and 0.3%, respectively, while 61.7% of the participants had no lipid abnormalities. High TG (individually, with other components being normal), high TC, low HDL, and high LDL were exclusively seen in 3.5%, 1.9%, 10.8%, and 2.3%, respectively. In general, most participants were aged 35–44 years (31.5%). They were mostly married (96.8%), and had < 6 years of education (71.9%). Most of them lived in urban areas (84.3%), had low socioeconomic status (44%), and were unemployed (84.8%). Moreover, 13.5% used hookah. With regard to medical history and anthropometric indices, 20.9% had diabetes, 30% had hypertension, most participants had BMI ≥ 25 kg/m^2^ (69.5%), 87.4% had high WHR, 76.4% had high WC based on the WHO cut-off, and 53.6% based on the Iranian-specific cut-off.

The prevalence of dyslipidemia and individual lipid abnormalities are demonstrated in Table 1. The prevalence of dyslipidemia, high TC, and high LDL was the highest in women aged 55–70 years, while high TG and low HDL were most prevalent in those aged 45–54 and 35–44 years, respectively. Except for high TG which was more prevalent among single women, dyslipidemia and all other lipid abnormalities were highest in married participants. Aside from low HDL, which was the highest in those with ≥ 6 years of education, dyslipidemia and all other lipid abnormalities were the highest in those with < 6 years of education. Apart from high TG and high TC, most prevalent in those living in rural areas, dyslipidemia and other lipid abnormalities were most prevalent in those living in urban areas. Details of lipid abnormalities are shown in Table 2.

**Table 1:** Prevalence of dyslipidemia and individual lipid abnormalities.

Variable		Total	Dyslipidemia	High TG	High TC	Low HDL	High LDL
		N (%)	N (%)	N (%)	N (%)	N (%)	N (%)
Age groups (years)	35–44	906 (40.8)	285 (31.5)	64 (7.1)	94 (10.4)	159 (17.5)	100 (11.0)
	45–54	711 (32.0)	300 (42.2)	98 (13.8)	155 (21.8)	94 (13.2)	143 (20.1)
	55–70	606 (27.3)	266 (43.9)	77 (12.7)	162 (26.7)	75 (12.4)	151 (24.9)
Marital status	Single	72 (3.2)	21 (29.2)	8 (11.1)	13 (18.1)	7 (9.7)	10 (13.9)
	Married	2151 (96.8)	830 (38.6)	231 (10.7)	398 (18.5)	321 (14.9)	384 (17.9)
Education	< 6 years	1598 (71.9)	653 (40.9)	182 (11.4)	333 (20.8)	226 (14.1)	324 (20.3)
	≥ 6 years	625 (28.1)	198 (31.7)	57 (9.1)	78 (12.5)	102 (16.3)	70 (11.2)
Place of residence	Urban	1874 (84.3)	730 (39.0)	197 (10.5)	340 (18.1)	296 (15.8)	340 (18.1)
	Rural	349 (15.7)	121 (34.7)	42 (12.0)	71 (20.3)	32 (9.2)	54 (15.5)
SES	Low	978 (44.0)	398 (40.7)	116 (11.9)	174 (17.8)	171 (17.5)	181 (18.5)
	Average	445 (20.0)	157 (35.3)	42 (9.4)	73 (16.4)	67 (15.1)	70 (15.7)
	High	800 (36.0)	296 (37.0)	81 (10.1)	164 (20.5)	90 (11.3)	143 (17.9)
Occupation	Unemployed	1884 (84.8)	733 (38.9)	205 (10.9)	361 (19.2)	280 (14.9)	343 (18.2)
	Employed	339 (15.2)	118 (34.8)	34 (10.0)	50 (14.7)	48 (14.2)	51 (15.0)
Hookah	No	1992 (86.5)	727 (37.8)	209 (10.9)	371 (19.3)	260 (13.5)	354 (18.4)
	Yes	301 (13.5)	124 (41.2)	30 (10.0)	40 (13.3)	68 (22.6)	40 (13.3)
Diabetes	No	1759 (79.1)	634 (36.0)	138 (7.8)	302 (17.2)	256 (14.6)	310 (17.6)
	Yes	464 (20.9)	217 (46.8)	101 (21.8)	109 (23.5)	72 (15.5)	84 (18.1)
Hypertension	No	1556 (70.0)	564 (36.2)	124 (8.0)	265 (17.0)	244 (15.7)	258 (16.6)
	Yes	667 (30.0)	287 (43.0)	115 (17.2)	146 (21.9)	84 (12.6)	136 (20.4)
BMI	< 25 kg/m^2^	677 (30.5)	221 (32.6)	46 (6.8)	110 (16.2)	85 (12.6)	114 (16.8)
	≥ 25 kg/m^2^	1546 (69.5)	630 (40.8)	193 (12.5)	301 (19.5)	243 (15.7)	280 (18.1)
WHO WC	Normal	525 (23.6)	152 (29.0)	30 (5.7)	72 (13.7)	61 (11.6)	75 (14.3)
	High	1698 (76.4)	699 (41.2)	209 (12.3)	339 (20.0)	267 (15.7)	319 (18.8)
Iranian WC	Normal	1031 (46.4)	353 (34.2)	82 (8.0)	178 (17.3)	129 (12.5)	182 (17.7)
	High	1192 (53.6)	498 (41.8)	157 (13.2)	233 (19.5)	199 (16.7)	212 (17.8)
WHO WHR	Normal	279 (12.6)	56 (20.1)	9 (3.2)	25 (9.0)	23 (8.2)	25 (9.0)
	High	1944 (87.4)	795 (40.9)	230 (11.8)	386 (19.9)	305 (15.7)	369 (19.0)

N, number; TG, triglyceride; TC, total cholesterol; HDL, high-density lipoprotein; LDL, low-density lipoprotein; SES, socioeconomic status; BMI, body mass index; WHO, World Health Organization; WC, waist circumference; WHR, waist-to-hip ratio.

**Table 2:** Details of lipid abnormalities based on ATP III cut-offs.

Variable	Range		N	%
TG	< 150 mg/dl	Normal	1641	73.8
	150–199 mg/dl	Borderline high	336	15.1
	200–499 mg/dl	High	240	10.8
	≥ 500 mg/dl	Very high	6	0.3
TC	< 200 mg/dl	Desirable	1025	46.1
	200–239 mg/dl	Borderline high	787	35.4
	≥ 240 mg/dl	High	411	18.5
HDL	< 50 mg/dl	Low	1129	50.8
	50–59 mg/dl	Normal	653	29.4
	≥ 60 mg/dl	High	441	19.8
LDL	< 100 mg/dl	Optimal	467	21.0
	100–129 mg/dl	Near or above optimal	688	30.9
	130–159 mg/dl	Borderline high	674	30.3
	160–189 mg/dl	High	272	12.2
	≥ 190 mg/dl	Very high	122	5.5

N, number; TG, triglyceride; TC, total cholesterol; HDL, high-density lipoprotein; LDL, low-density lipoprotein.

Logistic regression revealed that high WHR was consistently correlated with dyslipidemia and all of its components. Women living in urban areas were at significantly increased risk of dyslipidemia and low HDL compared to those living in rural areas, while ≥ 6 years of education was protective against high LDL. Age over 45 years was associated with dyslipidemia and high TC, while it was protective against low HDL. Women with diabetes and hypertension were at significantly increased risk of high TG; however, surprisingly hookah was protective against high TC and high LDL. Also, high socioeconomic status was protective regarding low HDL (Table 3).

**Table 3:** Logistic regression analysis of dyslipidemia and individual lipid abnormalities.

Variable		Dyslipidemia	High TG	High TC	Low HDL	High LDL
		OR (95% CI)	OR (95% CI)	OR (95% CI)	OR (95% CI)	OR (95% CI)
Age groups (years)	35–44					
	45–54	1.34 (1.07–1.68)*	1.57 (1.09–2.27)*	2.21 (1.63–2.99)*	0.68 (0.50–0.92)*	1.83 (1.36–2.47)
	55–70	1.33 (1.03–1.72)*	1.11 (0.73–1.69)	3.01 (2.16–4.19)*	0.59 (0.41–0.84)*	2.51 (1.81–3.49)
Marital status	Single					
	Married	1.08 (0.63–1.85)	0.68 (0.31–1.50)	0.63 (0.33–1.21)	1.48 (0.66–3.33)	0.89 (0.44–1.79)
Education	< 6 years					
	≥ 6 years	0.87 (0.69–1.09)	1.26 (0.87–1.81)	0.81 (0.60–1.10)	1.15 (0.86–1.55)	0.67 (0.49–0.92)*
Place of residence	Rural					
	Urban	1.35 (1.05–1.73)*	0.94 (0.65–1.38)	0.96 (0.71–1.29)	1.88 (1.26–2.79)*	1.38 (0.99–1.90)
SES	Low					
	Average	0.83 (0.65–1.06)	0.76 (0.51–1.11)	0.95 (0.70–1.29)	0.85 (0.62–1.17)	0.88 (0.64–1.19)
	High	0.88 (0.72–1.07)	0.79 (0.57–1.08)	1.21 (0.95–1.56)	0.60 (0.45–0.80)*	0.99 (0.77–1.29)
Occupation	Employed					
	Unemployed	1.04 (0.80–1.34)	0.89 (0.59–1.34)	0.93 (0.66–1.31)	0.86 (0.61–1.22)	0.99 (0.71–1.39)
Hookah	No					
	Yes	1.02 (0.79–1.32)	077 (051–1.17)	0.58 (0.41–0.84)*	1.71 (1.26–2.33)	0.60 (0.42–0.86)*
Diabetes	No					
	Yes	1.28 (1.00–1.60)	2.54 (1.87–3.46)*	1.09 (0.83–1.43)	1.20 (0.87–1.64)	0.76 (0.57–1.02)
Hypertension	No					
	Yes	0.99 (0.80–1.22)	1.62 (1.18–2.21)*	0.86 (0.67–1.12)	0.79 (0.58–1.07)	0.95 (0.73–1.23)
BMI	< 25 kg/m^2^					
	≥ 25 kg/m^2^	1.26 (0.98–1.62)	1.53 (0.99–2.35)	1.35 (0.99–1.86)	1.00 (0.70–1.44)	1.21 (0.88–1.66)
Iranian WC	Normal					
	High	0.98 (0.78–1.24)	1.02 (0.70–1.47)	0.80 (0.60–1.06)	1.32 (0.95–1.84)	0.76 (0.57–1.02)
WHO WHR	Normal					
	High	2.22 (1.60–3.08)*	2.58 (1.27–5.26)*	1.90 (1.21–2.99)*	2.19 (1.36–3.52)*	2.02 (1.28–3.18)*

OR, odds ratio; CI, confidence interval; TG, triglyceride; TC, total cholesterol; HDL, high-density lipoprotein; LDL, low-density lipoprotein; SES, socioeconomic status; BMI, body mass index; WHO, World Health Organization; WC, waist circumference; WHR, waist-to-hip ratio.

*Statistically significant (*P*-value < 0.05).

**Figure 1:**
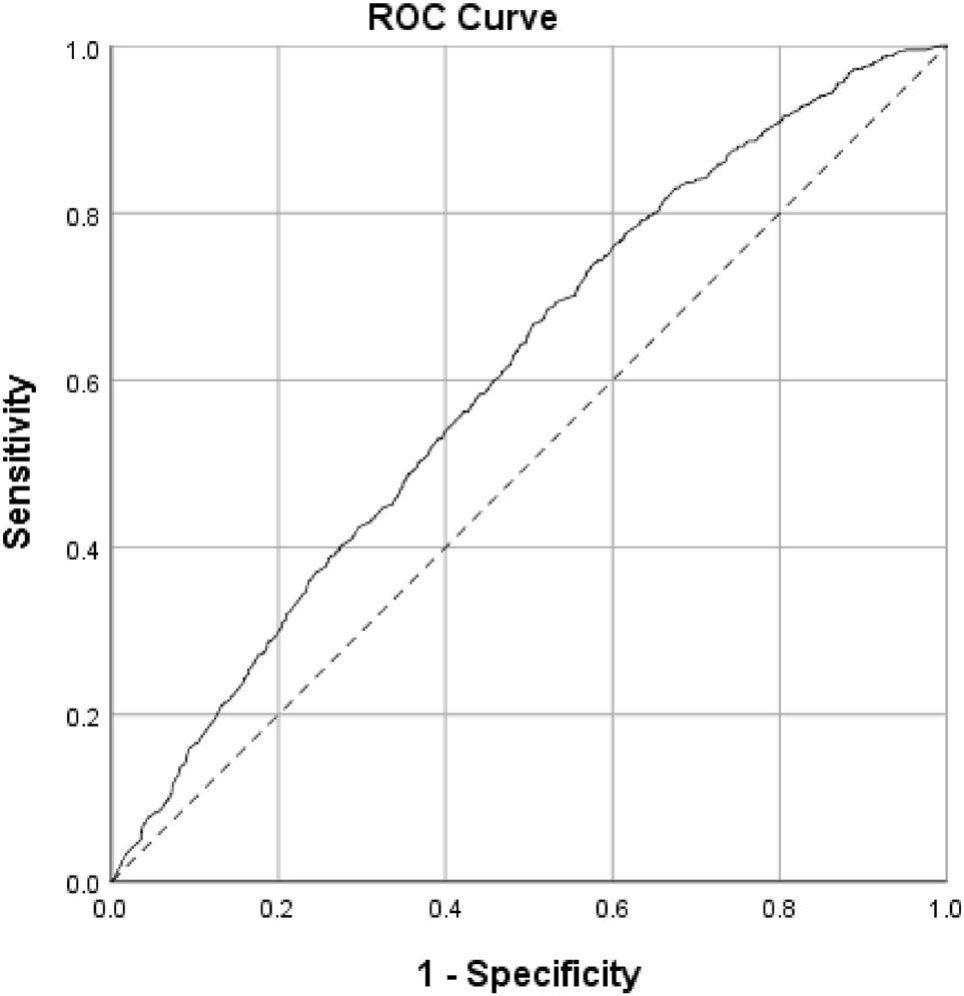
Receiver operating characteristic (ROC) curve for prediction of dyslipidemia by the logistic regression model.

The AUROC curve of the logistic regression model for prediction of dyslipidemia was 0.607 (95% CI 0.583–0.630) which shows the relatively acceptable performance of this model (61%) (Fig. 1).

Linear regression ("enter" method) showed that every 1 kg/m^2^ increase in BMI led to 2.51, 1.56, and 0.94 mg/dl increase in TG, TC, and LDL, respectively. Every 1 mg/dl increase in FPG and every 1 mmHg increase in systolic blood pressure (SBP) led to 0.22 and 0.41 mg/dl increase in TG, respectively. Every 1 year advance in age and 1 mmHg elevation in diastolic blood pressure (DBP) led to 0.59 and 0.31 mg/dl increase in TC, respectively. Weight, HC, and WHR were negatively correlated with HDL. Age was also positively associated with LDL. Besides, altogether age, years of education, weight, WC, HC, WHR, BMI, FPG, SBP, DBP, daily calorie intake, and weekly METs could predict 14%, 6%, 7%, and 3% of the variance of TG, TC, HDL, and LDL, respectively (Table 4).

**Table 4:** Multiple regression analysis of predictors of lipid profile components.

Dependent variable	Predictors	B	B (95% CI)	*P*-value	Adjusted R^2^
TG	Age	0.269	− 0.127 to 0.665	0.182	0.137 (*P*-value < 0.001)
	Years of education	0.244	− 0.482 to 0.971	0.510	
	Weight	1.113	0.487 to 1.739	< 0.001	
	WC	− 2.905	− 6.632 to 0.822	0.127	
	HC	0.623	− 3.021 to 4.267	0.737	
	WHR	382.448	− 4.594 to 769.491	0.053	
	BMI	2.511	0.922 to 4.100	0.002	
	FPG	0.228	0.166 to 0.290	< 0.001	
	SBP	0.416	0.143 to 0.688	0.003	
	DBP	− 0.041	− 0.487 to 0.404	0.856	
	Daily calorie intake	0.004	0.001 to 0.008	0.024	
	Weekly METs	− 0.058	− 0.143 to 0.027	0.182	
TC	Age	0.598	0.342 to 0.854	< 0.001	0.059 (*P*-value < 0.001)
	Years of education	0.001	− 0.469 to 0.470	0.998	
	Weight	− 0.425	− 0.829 to − 0.021	0.039	
	WC	0.083	− 2.325 to 2.491	0.946	
	HC	− 0.135	− 2.489 to 2.219	0.910	
	WHR	− 0.935	− 250.971 to 249.100	0.994	
	BMI	1.564	0.537 to 2.590	0.003	
	FPG	0.035	− 0.005 to 0.075	0.084	
	SBP	0.079	− 0.097 to 0.255	0.381	
	DBP	0.318	0.030 to 0.606	0.030	
	Daily calorie intake	0.001	− 0.001 to 0.004	0.236	
	Weekly METs	0.086	0.031 to 0.141	0.002	
HDL	Age	0.171	0.106 to 0.235	< 0.001	0.072 (*P*-value < 0.001)
	Years of education	0.117	− 0.002 to 0.236	0.054	
	Weight	− 0.300	− 0.402 to − 0.198	< 0.001	
	WC	1.294	0.685 to 1.904	< 0.001	
	HC	− 0.883	− 1.478 to − 0.287	0.004	
	WHR	− 148.504	− 211.774 to − 85.233	< 0.001	
	BMI	0.022	− 0.238 to 0.281	0.870	
	FPG	0.011	0.001 to 0.021	0.034	
	SBP	− 0.013	− 0.058 to 0.031	0.559	
	DBP	0.055	− 0.018 to 0.128	0.138	
	Daily calorie intake	< 0.001	− 0.001 to 0.001	0.794	
	Weekly METs	0.018	0.004 to 0.032	0.010	
LDL	Age	0.358	0.141 to 0.574	0.001	0.031 (*P*-value < 0.001)
	Years of education	− 0.179	− 0.577 to 0.219	0.379	
	Weight	− 0.310	− 0.653 to 0.032	0.076	
	WC	− 0.742	− 2.783 to 1.300	0.476	
	HC	0.720	− 1.276 to 2.716	0.479	
	WHR	83.109	− 128.874 to 295.092	0.442	
	BMI	0.945	0.075 to 1.815	0.033	
	FPG	− 0.014	− 0.48 to 0.020	0.414	
	SBP	0.045	− 0.104 to 0.195	0.552	
	DBP	0.230	− 0.014 to 0.474	0.064	
	Daily calorie intake	0.001	− 0.001 to 0.003	0.480	
	Weekly METs	0.080	0.033 to 0.126	0.001	

TG, triglyceride; TC, total cholesterol; HDL, high-density lipoprotein; LDL, low-density lipoprotein; WC, waist circumference; HC, hip circumference; WHR, waist-to-hip ratio; BMI, body mass index; FPG, fasting plasma glucose; SBP, systolic blood pressure; DBP, diastolic blood pressure; MET, metabolic equivalent of task; CI, confidence interval.

The group correlation between independent and effective variables with dyslipidemia (dependent variable) was evaluated using deep learning. It is possible to completely assess the singular correlation between the mentioned variables through statistical analyses that have been performed previously. The model consists of a deep learning feed forward network with 9 layers as follows:

Model: "Sequential".

**Table:** 

Layer (type)	Output shape	Parameters
dense (Dense)	(None, 64)	4032
dense_1 (Dense)	(None, 32)	2080
dense_2 (Dense)	(None, 64)	2112
dense_3 (Dense)	(None, 32)	2080
dense_4 (Dense)	(None, 64)	2112
dropout (Dropout)	(None, 64)	0
batch_normalization	(None, 64)	256
dense_5 (Dense)	(None, 2)	130

Total parameters: 12,802, Trainable parameters: 12,674, Non-trainable parameters: 128.

As shown above, the model consists of 9 layers including the input layer, and 12,802 parameters, of which 12,674 parameters were trainable. In each layer, the number of neural network nodes and parameters of that layer has been expressed. The network with variety of variables was evaluated. We reached the best performance in accuracy and predictability for the following variables (age groups ≥ 45 years, urban residence, and high WHR). The resulting confusion table is based on 304 negative samples and 244 positive samples as follows:

**Table:** 

		Decision	
		No dyslipidemia	Dyslipidemia
Target	No dyslipidemia	201	103
	Dyslipidemia	86	158

The overall precision of the model is equal to 0.65% in the considered range.

**Table:** 

Class	Precision	Recall	F1-score	Support
No dyslipidemia	0.7	0.66	0.68	304
Dyslipidemia	0.61	0.65	0.63	244

The average performance of the model is:

**Table:** 

		Decision	
		No dyslipidemia	Dyslipidemia
Target	No dyslipidemia	261	141
	Dyslipidemia	158	107

and the mean's precision is 0.55%.

**Table:** 

Class	Precision	Recall	F1-score	Support
No dyslipidemia	0.62	0.65	0.64	402
Dyslipidemia	0.43	0.4	0.42	265

Therefore, the model confirmed 45% of the cases in the target range with 65% of precision. This means a definite confirmation of 35% of cases in the database. The model also confirms 40% of cases in the entire database with 55% precision. This means a definite confirmation of 22% of cases in the database. By comparing the two results, it could be concluded that the risk of dyslipidemia is higher in those older than 45 years, urban dwellers, and individuals with central obesity.

## Discussion

The primary finding of the current study was the high prevalence of dyslipidemia in women aged 35–70 years of the PERSIAN Bandare Kong Cohort Study, with 38.3% of the study population having at least one lipid abnormality. High TC was the most common, (18.5%) followed by high LDL (17.7%), low HDL (14.8%), and high TG (10.8%).

Prevalence of dyslipidemia in women was 87.7% in Najafipour et al.'s study^23^, 61.3% in the study by Ebrahimi et al.^24^, 85.1% in Latifi et al.'s study^25^, and 37.6% among Chinese women^2^. In a systematic review and meta-analysis on the prevalence of dyslipidemia in published articles in Iran until September 2011, hypercholesterolemia, low HDL, and high LDL were more prevalent in women compared to men^26^. Another study showed that the prevalence of hypertriglyceridemia and hypercholesterolemia were 33.2% and 45.4% among females^27^. The corresponding percentages were 41% and 23% in Japanese women aged 25–64 years^28^, 25% and 37.2% in Turkish adults^29^, 24.1% and 30.6% in Najafipour et al.'s study^23^, and 65.1% and 47.5% in Latifi et al.'s study^25^. The variety of the prevalence of dyslipidemia and its individual components across different studies can be explained by demographic, socioeconomic, and anthropometric features of study populations which will be discussed in detail later on.

The results of this study showed that the prevalence of dyslipidemia steadily increased with age in women. A similar trend was observed in Ebrahimi et al.'s study^24^. Nonetheless, we found that the odds of dyslipidemia in women aged 45–54 and 55–70 years were similar based on the logistic regression analysis. Yet, other studies in different parts of the world have shown that the risk of different types of dyslipidemia increases with age in both men and women^29–31^.

We found no correlation between marital status and dyslipidemia or any of its components, which was in line with the findings of Ebrahimi et al.^24^. While high TG was more prevalent among single women, dyslipidemia and all other lipid abnormalities were highest in married participants in our study. Whereas, aside from low HDL, other lipid abnormalities were higher in married and widowed individuals compared to singles in the study by Erem et al.^29^.

According to the findings of the current study, ≥ 6 years of education was protective against high LDL; however, level of education was not associated with dyslipidemia and other lipid abnormalities. Quite similarly, dyslipidemia was not influenced by level of education in a study conducted in India^11^. In addition, this was partially consistent with the results of Ebrahimi et al.'s study, in which no relationship was found between the risk of developing different types of dyslipidemia and the level of education^24^. On the contrary, Erem et al. in their study to estimate the prevalence of dyslipidemia and associated factors among Turkish adults, demonstrated that the risk of dyslipidemia was higher in those with lower education level. They justified their findings by higher exposure to risk factors such as poor eating habits and working conditions, difficulty to access health services, and stress in those with low level of education^29^. Of note, the findings of the above-mentioned studies were with respect to both men and women. Results can be different when only women are concerned.

We found that women living in living in rural areas were at significantly increased risk of dyslipidemia and low HDL compared to those living in urban areas. On the contrary, the odds of hypertriglyceridemia and hypercholesterolemia, although insignificant, was lower in women living in urban areas. Similarly, Cui et al. demonstrated that the prevalence of hypercholesterolemia was higher in rural areas compared to urban areas for women^28^. Contrarily, Tripathy et al. demonstrated that living in rural areas increased the risk of dyslipidemia and high TC^32^. The reason for these inconsistencies may be the consumption of fat-rich foods such as high-fat dairy by individuals living in rural areas in the two studies.

Over the past decade, there has been a decline in consumption of traditional foods, while use of high-fat, high-calorie, low-fiber, and processed foods has increased. This unhealthy diet together with insufficient physical activity, are risk factors for obesity and hypertriglyceridemia^33–35^. Among the participants of the current study, 69.5% were overweight or obese. Overweight and obesity were not correlated with dyslipidemia or individual lipid abnormalities. Among the anthropometric indices, high WHR was the best predictor of dyslipidemia and all of its components. Obesity has been reported to be associated with hypertriglyceridemia in many studies^8, 11, 29–31, 36, 37^. It should be noted that contrary to our findings, obesity has been proposed as a risk for hypercholesterolemia in some studies^8, 11, 29, 36^.

The ROC curve predicted the logistic regression model's performance once with all the variables included in the model and then with the significant factors (high WHR, age over 45 years, and living in urban areas). The AUROC of the model for significant factors was 0.61. Besides, based on our findings in the deep learning models, the accuracy was 65%. The accuracy of the prediction model is lower than our expectation which might be explained by the effect of unknown variables that have not been measured in the cohort study.

The odds of hypertriglyceridemia was significantly higher in women with hypertension or diabetes in our study, while neither dyslipidemia nor any other components were associated with the two comorbidities. This was in agreement with the findings of Ebrahimi et al.; however, they also reported a significant correlation between high blood pressure and dyslipidemia^24^. Tabrizi et al. reported similar results^38^. Despite comparable findings regarding the association between hypertension and hypertriglyceridemia in a study in India, dyslipidemia and other lipid abnormalities were also significantly correlated in this study^32^. Lipids are the major components of atherosclerotic plaques which are associated with hypertension through a decrease in the vascular lumen diameter and an increase in the arterial wall resistance; moreover, dyslipidemia can cause endothelial damage leading to the disturbance of the physiological vasomotor activity^39^. As for the relationship between dyslipidemia and diabetes, the effect of insulin resistance on key enzymes involved in lipid metabolism has been established resulting in diabetic dyslipidemia consisting of a triad of increased LDL, decreased HDL, and raised triglycerides^40^.

One limitation of the current study was that although, lipid-lowering medications were taken into account, some women with diabetes may have failed to indicate that they were taking these agents, which resulted in contradictory findings regarding the relationship between dyslipidemia or its components with diabetes. Another limitation was the assessment of physical activity which was reported in METs. The positive effect of physical activity on serum lipids, plasma glucose, and many other CVD risk factors has been previously established; therefore, the increase in TC and LDL with higher weekly METs observed in the current study can in part be due to inaccurate evaluation of physical activity. One more limitation was the daily calorie intake that was not subdivided based on specific foods. Calorie content of fat-rich foods would have been more valuable in the assessment of the correlation between lipid abnormalities and daily calorie intake.

## Conclusions

Dyslipidemia was highly prevalent in women of the PERSIAN Bandare Kong Cohort Study. High TC was the most common, and high TG the least common lipid abnormalities in this population. High WHR put women aged 35–70 years at high risk of dyslipidemia and all of its components and appears to be the best predictive anthropometric index with regard to lipid abnormalities. Women over 45 years were at highest risk of developing lipid abnormalities compared to other age groups. Living in urban areas positively influenced the lipid profile of women, while marital status and employment had no effect on it. Except for the positive effect of ≥ 6 years of education on high LDL, level of education did not affect lipid abnormalities. Although the odds of high TG was higher in those with hypertension or diabetes. The significance of hookah use for lipid abnormalities was paradoxical. These findings should be taken into consideration in the preparation of future management and prevention guidelines designated for this specific population. Future studies are needed to identify unknown variables in the prediction of dyslipidemia.

## Data Availability

The datasets used and/or analyzed during the current study are available from the corresponding author on reasonable request.

## Abbreviations

ADA: American Diabetes Association
ATP III: Adult Treatment Panel III
AUROC: Area under the receiver operating characteristic (curve)
BMI: Body mass index
BP: Blood pressure
CI: Confidence interval
CVD: Cardiovascular disease
DALYS: Disability-adjusted life years
DBP: Diastolic blood pressure
FPG: Fasting plasma glucose
HC: Hip circumference
HDL: High-density lipoprotein
LDL: Low-density lipoprotein
MET: Metabolic equivalent of task
OR: Odds ratio
PERSIAN: Prospective Epidemiological Research Studies in IrAN
ROC: Receiver operating characteristic
SBP: Systolic blood pressure
SES: Socioeconomic status
SPSS: Statistical Package for the Social Sciences
TC: Total cholesterol
TG: Triglyceride
WC: Waist circumference
WHO: World Health Organization
WHR: Waist-to-hip ratio
